# Supplementary material for: Modelling Metabolic Shifts during Cardiomyocyte Differentiation, Iron Deficiency and Transferrin Rescue Using Human Pluripotent Stem Cells
Source: Metabolites. 2021 Dec 22;12(1):9. doi: 10.3390/metabo12010009 (PMC8778576; doi:10.3390/metabo12010009)
Supplement: Supplementary file 1 [file metabolites-12-00009-s001.zip › metabolites-1461641-supplementary.pdf]

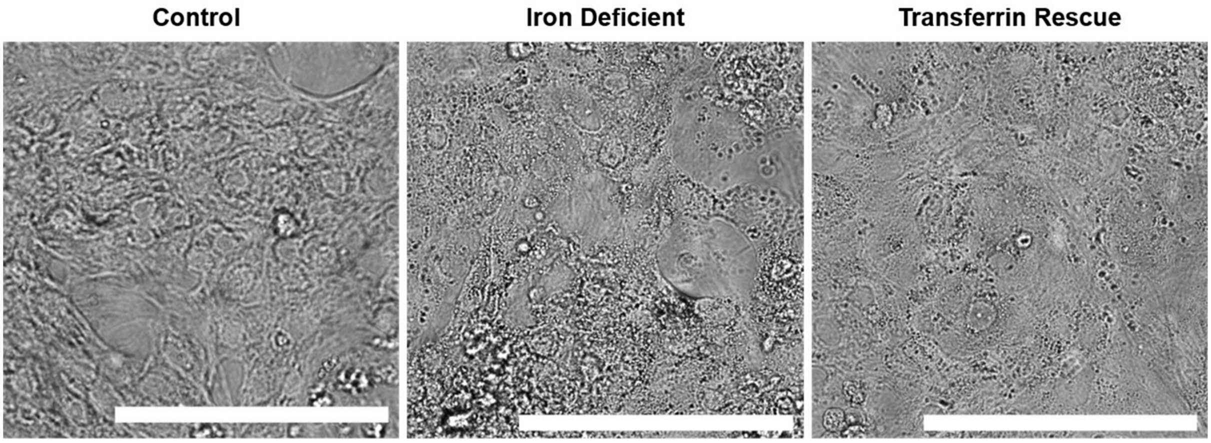

Supplementary Figure S1. Bright field images of morphological changes of hiPSC-CMs during iron deficiency and transferrin rescue.

Images captured on EVOS M5000. Scale bar at 125 µm.
